# Assessment Methods of Body Fat in Recreational Marathon Runners: Bioelectrical Impedance Analysis versus Skinfold Thickness

**DOI:** 10.1155/2021/3717562

**Published:** 2021-09-29

**Authors:** Pantelis T. Nikolaidis, Rodrigo Luiz Vancini, Marília dos Santos Andrade, Claudio Andre Barbosa de Lira, Beat Knechtle

**Affiliations:** ^1^School of Health and Caring Sciences, University of West Attica, Egaleo, Greece; ^2^Exercise Physiology Laboratory, Nikaia, Greece; ^3^Centro de Educação Física e Desportos (CEFD), Universidade Federal do Espírito Santo (UFES), Espírito Santo (ES), Brazil; ^4^Departamento de Fisiologia, Universidade Federal de São Paulo (UNIFESP), São Paulo (SP), Brazil; ^5^Setor de Fisiologia Humana e do Exercício, Faculdade de Educação Física e Dança, Universidade Federal de Goiás (UFG), Goiânia, Goiás (GO), Brazil; ^6^Medbase St. Gallen Am Vadianplatz, St. Gallen, Switzerland; ^7^Institute of Primary Care, University of Zurich, Zurich, Switzerland

## Abstract

The aim of the present study was to examine (a) the relationship of body fat (BF) assessed by bioimpedance analysis (BIA) and skinfold thickness (SKF) and (b) the variation of BF by age depending on the assessment method. Participants were 32 women and 134 men recreational marathon runners, who were tested for BF using both assessment methods (BIA and SKF). Rc between BIA and SKF assessment methods was 0.803 (95% CI; 0.640, 0.897) in women and 0.568 (95% CI; 0.481, 0.644) in men. A large main effect of the assessment method on BF was observed (*p* < 0.001, *η*^2^ = 0.156) with SKF presenting higher BF than BIA by 2.9%. The difference between SKF and BIA was 3.9 ± 2.7% (95% confidence intervals, CI; 3.4; 4.3, *p* < 0.001) in men, whereas no difference was found in women (−0.9 ± 2.9%; 95% CI; -1.9; -0.2, *p* = 0.101). BF correlated with age with small magnitude (BIA, *r* = 0.18, *p* = 0.036; SKF, *r* = 0.23, *p* = 0.007) in men, i.e., the older the age, the higher the BF. A similar trend of moderate magnitude was observed in women for BIA (*r* = 0.45, *p* = 0.011), but not for SKF (*r* = 0.33, *p* = 0.067). In conclusion, practitioners involved in the training of recreational runners would be advised to consider that BIA elicits a lower BF value than the SKF method in men.

## 1. Introduction

Body fat percentage (BF) has been a predictor of race time in marathon runners—with faster times associated with lower BF—and has been routinely monitored in this sport [1–3]. For instance, elite marathon runners with a personal record less than 2 h 10 min were characterized by low BF (<10%) [4], whereas recreational runners with a personal record close to 4 h had BF higher than 15% [5]. An explanation of this relationship of BF with performance might be that adipose tissue consisted of an excess mass that runners should carry across a long distance increasing the metabolic demands [6]. BF has been widely assessed in marathon runners using skinfold thickness (SKF) in a certain number of anatomical sites, e.g., seven [4, 7] and ten [8] sites. In addition to SKF, BF has also been evaluated using the technique of bioelectrical impedance analysis (BIA) [9].

Little information existed so far about the relationship between measures of BF relying on SKF and BIA in recreational runners. Considering the popularity of SKF [2, 10] and BIA [11, 12] to evaluate BF and that these methods might be used interchangeably in the context of training monitoring and performance prediction [13], it would be of practical importance for marathon runners and practitioners working with them to be aware of the relationship between SKF and BIA.

A comparison of these measures in ultraendurance swimmers, cyclists, and triathletes indicated higher values of BF in BIA than in SKF [14]. Since both methodological approaches were widely applied in marathon runners [4, 9], knowledge on the relationship between these approaches could provide valuable information to professionals (e.g., exercise physiologists, sport nutritionists, coaches, and trainers); it would be of great practical importance for practitioners to optimize body mass and body composition of their runners. Studying the relationship between BIA and SKF would concern a large number of athletes—of both sexes and of a wide range of age—who might use these assessment methods interchangeably [15]. Thus, the aim of the present research was to investigate (a) the relationship of BF assessed by BIA and SKF and (b) the variation of BF by age and BMI depending on the assessment method.

## 2. Materials and Methods

### 2.1. Participants

Participants were 32 women (age, 40 ± 9 years, height 162 ± 7 cm, and body mass 58 ± 8 kg) and 134 men (44 ± 9 years, 176 ± 6 cm, and 77 ± 9 kg, respectively) marathon runners, who had responded to a public call through social media. All participants finished the Athens marathon 2017. After having been informed about the procedures of the research, all participants provided written informed consent. Female and male participants had record in marathon race time 4 : 34 ± 0 : 39 and 4 : 02 ± 0 : 44 h:min, experience in running training 5.5 ± 4.6 and 6.8 ± 5.8 years, 3.3 ± 3.6 and 5.6 ± 6.3 finished marathons, weekly training days 4.1 ± 1.5 and 4.4 ± 1.2, and weekly training running distance 47.7 ± 22.6 and 53.2 ± 21.1 km, respectively.

### 2.2. Procedures

Height and weight were measured with subjects in minimal clothing and barefoot. A weighing scale HD-351 (Tanita, Arlington Heights, IL, USA) was used for measurement of weight (to the nearest 0.1 kg) and a portable stadiometer (SECA, Leicester, UK) for height (to the nearest 0.1 cm). The thickness of ten SKF (cheek, chin, pectoral, triceps, subscapular, abdomen, chest II, iliac crest, patella, and proximal calf) was measured to the nearest 0.1 mm (Harpenden, West Sussex, UK) [16]. Parizkova's equations (women, BF = 39.572 log*X* − 61.25; men, BF = 22.32 log*X* − 29; *X* = sum of 10 SKF) were used to estimate BF [16]. An exercise physiologist with experience of measuring SKF with an intraclass correlation coefficient higher than 0.99 in over 10,000 subjects performed all assessments including anthropometric characteristics. Considering the excellent intraclass coefficient correlation of the tester, a single assessment was performed for each anatomical site of SKF. Moreover, BF was evaluated by Tanita BC-545 BIA (Tanita, Arlington Heights, IL, USA). Prior to BIA measurement, sex, age, height, and training status (i.e., being athlete or not) were entered in Tanita BC-545. With regard to the Tanita BC-545 BIA's option of “being athlete or not,” the athlete mode was selected for all participants, and consequently, the corresponding training-specific in-built prediction equations were applied. All testing procedures were carried out on a single session and in the same order (height, weight, SKF, and BIA) by the same researcher who had large experience in the assessment of anthropometry and body composition.

### 2.3. Statistical and Data Analysis

All analyses were performed by GraphPad Prism v. 7.0 (GraphPad Software, San Diego, USA) and IBM SPSS v.26.0 (SPSS, Chicago, USA). Statistical significance was set at alpha = 0.05. Data were presented as the mean and standard deviations. Lin's concordance correlation coefficient (Rc) examined the relationship between BIA and SKF assessment methods, and 95% confidence intervals (CI) were calculated. A between-within analysis of variance (ANOVA) examined the main effects of assessment method (BIA versus SKF) and sex, and their interaction on BF, and eta squared (*η*^2^) estimated the magnitude of differences. Bland-Altman plots were used to analyze the agreement between SKF and BIA method to assess BF, and Pearson correlation coefficient *r* examined the relationship between the difference (SKF minus BIA) and average values ((SKF + BIA)/2) for each sex.

## 3. Results

**Figure 1:**
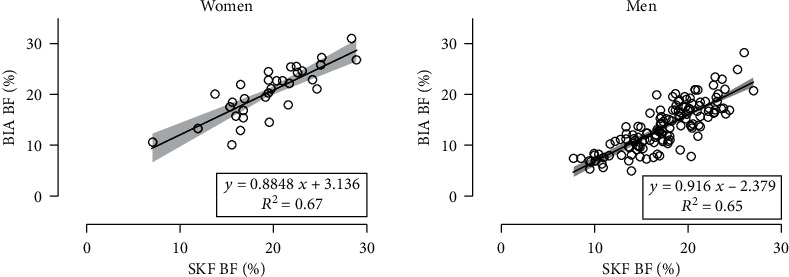
Relationship of body fat percentage (BF) estimated by bioelectrical impedance analysis (BIA) and skinfold (SKF) method. *R*^2^ = coefficient of determination.

Rc between BIA and SKF assessment methods was 0.803 (95% CI; 0.640, 0.897) in women and 0.568 (95% CI; 0.481, 0.644) in men (Figure 1). In women, SKF BF and BIA BF did correlate with any training variable (∣*r* | ≤0.22, *p* ≥ 0.225). In men, SKF BF and BIA BF correlated with weekly training days (*r* = −0.39, *p* < 0.001; *r* = −0.32, *p* < 0.001, respectively) and weekly running distance (*r* = −0.41, *p* < 0.001; *r* = −0.35, *p* < 0.001, respectively), but not with the number of finished marathons or years of running training (∣*r* | ≤0.14, *p* ≥ 0.116).

The between-within subjects ANOVA showed a large main effect of the assessment method on BF (*p* < 0.001, *η*^2^ = 0.156) with overall SKF value being higher than BIA by 2.9% (Table 1). A large sex×assessment method interaction on BF was observed (*p* < 0.001, *η*^2^ = 0.317) with the difference between SKF's and BIA's BF being higher in men than in women. Particularly, a paired-samples *t*-test showed that this difference was 3.9 ± 2.7% (95% confidence intervals, CI; 3.4; 4.3, *p* < 0.001) in men, whereas no difference was found in women (−0.9 ± 2.9%; 95% CI; -1.9; -0.2, *p* = 0.101).

**Figure 2:**
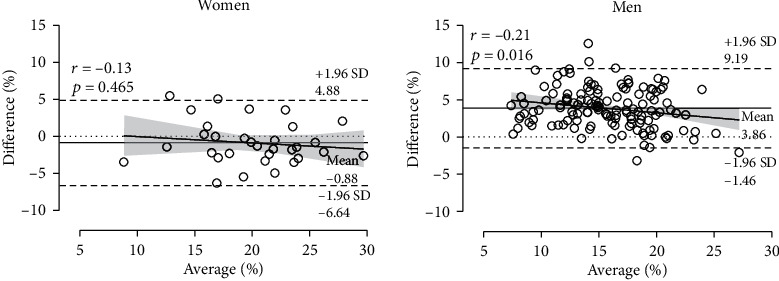
Bland-Altman plots showing the difference (bias) between skinfold thickness (SKF) and bioelectrical impedance analysis' (BIA) body fat percentage (BF) (average). Difference = SKF_BF–BIA_BF; average = (SKF_BF + BIA_BF)/2.

**Figure 3:**
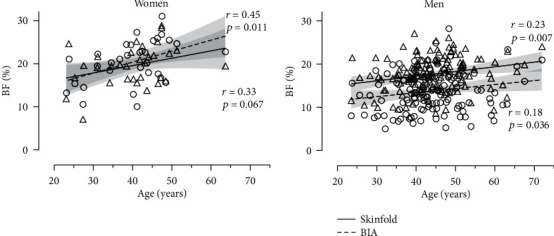
Body fat percentage (BF) assessed by skinfold and BIA methods in relation to age.

An analysis of Bland-Altman plots indicated a negative relationship between the difference between the two assessment methods and their average value of small magnitude, i.e., the larger the BF, the smaller their difference (Figure 2). BF correlated with age with small magnitude (BIA, *r* = 0.18, *p* = 0.036; SKF, *r* = 0.23, *p* = 0.007) in men, i.e., the older the age, the higher the BF. A similar trend of moderate magnitude was observed in women for BIA (*r* = 0.45, *p* = 0.011), but not for SKF (*r* = 0.33, *p* = 0.067) (Figure 3). The difference between BIA and SKF BF did not correlate with age in women (*r* = −0.25, *p* = 0.176) and men (*r* = 0.04, *p* = 0.667), but it correlated with BMI in men (*r* = −0.26, *p* = 0.002; women, *r* = −0.28, *p* = 0.117).

## 4. Discussion

The main findings of the present research were that (a) BIA and SKF provided comparable BF in women but differed in men (lower BF in BIA than SKF by ~4%) and (b) age correlated low-to-moderately with BF depending on sex and assessment method. With regard to the role of age in BF, the small-to-moderate magnitude of this relationship might be attributed to long-term adaptations to exercise resulting in attenuation of the increase of BF with age. A recent study showed that men runners ~50 years old had less BMI and BF than a control group [17]. Also, it was found previously that the differences between younger and elder men were greater for visceral fat than for subcutaneous fat [18]. That is, the aging process per se as well as the increase in BF with age can be a key factor in sports performance [19–22]. In addition, the discrepancy between BIA and SKF found in women was in agreement with a previous study [23] confirming a tendency for higher BF in BIA than in SKF with aging.

The evaluation of BIA and SKF elicited comparable values in women, but different in men, where BIA provided the lowest value. The discrepancy of these methods in men should be attributed to variations in hydration status [24] as it has been observed that hydration levels impacted BIA BF [25]. In addition, the reliability of BIA might depend on factors linked to the apparatus, e.g., electrodes and participants [26]. Through Bland-Altman analysis, it was possible to observe that the SKF and BIA methods were more divergent in individuals presenting low fat mass percentage than in individuals presenting high fat mass. This might justify why the women, who traditionally presented higher BF than men, had lower divergence between the two ways of estimating BF than the men. This observation was in line with a comparison between BIA and SKF in older adults, where women had higher BF than men with both assessment methods and showed a better level of agreement between the methods [27].

The lower value by ~4% of men's BF in BIA than in the SKF method in the present study was in line with research reporting comparable differences in soldiers (~3.5%) [28] and physically active adults (~5.5%) [29], whereas a smaller difference was observed in hikers [30]. A common characteristic of the studies reporting lower BF in BIA than in SKF was that they were conducted on physically active men or athletes [28–30]. On the other hand, there were studies reporting higher BF in BIA than in SKF [31, 32]. For instance, BIA elicited ~6% higher BF than seven-site SKF in young adults [31] and ~2.5% higher BF than SKF in hemodialysis patients [32].

With regard to the limitations of the specific methods of BF evaluation, it was recognized that there were other SKF methods—based on different equations and/or number of anatomical sites—that could elicit different values of BF [33]; similarly, other BIA devices might also differ for the estimation of BF [34] considering the specific prediction algorithms and the technical characteristics of the device. Therefore, the results of this research should be generalized carefully to other assessment tests of body composition. Another limitation of the study might be the unequal sample sizes between female and male participants; however, the variance of values—as indicated by standard deviation of both methods' BF—did not differ by sex suggesting that the assumption of equal variances in ANOVA was not violated. Furthermore, it should be highlighted that the smaller sample size of female than male participants (corresponding to a men-to-women ratio 4.19) was ecologically valid since it reflected the men-to-women ratio observed in several marathon races (e.g., 2.36 in the New York City marathon 1970-2017 [35], 3.86 in the Oslo marathon 2008-2018 [36], and 4.06 in the Athens marathon 2017 [37]). The results might provide practical information to monitor training in view of the popularity of marathon races [35, 38] and the impact of BF on running performance [2, 39]. However, it should be highlighted that—since neither the one nor the other of the two methods was considered a golden standard of body composition—the present study concerned the comparison of two indirect assessment methods of body fat (SKF and BIA) [40, 41] and was not aimed at validating the one using the other one as reference.

It should be noted that the abovementioned discussion focused on the consideration of mean scores. In this context, the findings of the present study would have practical applications for coaches and fitness trainers working with a group of endurance runners rather than working with a single runner. An analysis of Bland-Altman plots (Figure 2), where the limits of agreement (LoA) provided a measure of agreement at an individual level (i.e., a single runner), showed a relatively large level of agreement (LoA -6.64 to 4.88% in women and -1.46 to 9.19% in men) precluding interchangeability of the two assessment methods at an individual level.

In conclusion, professionals working with recreational runners should consider that bioelectrical impedance analysis might provide lower body fat percentage scores than the SKF method in men. Therefore, we suggest that practitioners avoid using mixed devices to monitor the effects of training intervention on BF.

## Figures and Tables

**Table 1:** Body fat percentage and body mass index by sex.

Variable	Women (*n* = 32)	Men (*n* = 134)
BMI (kg·m^−2^)	21.8 ± 2.2	24.7 ± 2.6^∗∗^
SKF_BF (%)	19.6 ± 4.7	17.7 ± 4.0^∗^
BIA_BF (%)	20.5 ± 5.0	13.8 ± 4.6^∗∗^

BMI = body mass index; BF = body fat; SKF = skinfold thickness; BIA = bioelectrical impedance analysis; sex difference at ^∗^*p* < 0.05 and ^∗∗^*p* < 0.001.

## Data Availability

All data are available by P.N. upon reasonable request.

## References

[1] Alvero-Cruz J. R., Carnero E. A., García M. A. G. (2020). Predictive performance models in long-distance runners: a narrative review. *International Journal of Environmental Research and Public Health*.

[2] Salinero J. J., Soriano M. L., Lara B. (2017). Predicting race time in male amateur marathon runners. *The Journal of sports medicine and physical fitness*.

[3] Knechtle B., Tanda G. (2015). Effects of training and anthropometric factors on marathon and 100 km ultramarathon race performance. *Open access Journal of Sports Medicine*.

[4] Vernillo G., Schena F., Berardelli C. (2013). Anthropometric characteristics of top-class Kenyan marathon runners. *The Journal of Sports Medicine and Physical Fitness*.

[5] Zillmann T., Knechtle B., Rüst C. A., Knechtle P., Rosemann T., Lepers R. (2013). Comparison of training and anthropometric characteristics between recreational male half-marathoners and marathoners. *The Chinese Journal of Physiology*.

[6] Lukaski H., Raymond-Pope C. J. (2021). New frontiers of body composition in sport. *International Journal of Sports Medicine*.

[7] Knechtle B., Tanda G. (2013). Marathon performance in relation to body fat percentage and training indices in recreational male runners. *Open access Journal of Sports Medicine*.

[8] Nikolaidis P. T., Knechtle B. (2018). Pacing strategies in the 'Athens classic marathon': physiological and psychological aspects. *Frontiers in Physiology*.

[9] Marra M., Di Gregorio A., Alicante P. Evaluation of body composition in competitive male marathon runners.

[10] Ogueta-Alday A., Morante J. C., Gómez-Molina J., García-López J. (2018). Similarities and differences among half-marathon runners according to their performance level. *PLoS One*.

[11] Hottenrott K., Ludyga S., Schulze S. (2012). Effects of high intensity training and continuous endurance training on aerobic capacity and body composition in recreationally active runners. *Journal of sports science & medicine*.

[12] Nikolaidis P. T., Knechtle C., Ramirez-Campillo R., Vancini R. L., Rosemann T., Knechtle B. (2019). Training and body composition during preparation for a 48-hour ultra-marathon race: a case study of a master athlete. *International Journal of Environmental Research and Public Health*.

[13] Schütz U. H., Schmidt-Trucksäss A., Knechtle B. (2012). The Transeurope FootRace Project: longitudinal data acquisition in a cluster randomized mobile MRI observational cohort study on 44 endurance runners at a 64-stage 4,486km transcontinental ultramarathon. *BMC Medicine*.

[14] Knechtle B., Wirth A., Knechtle P., Rosemann T., Rust C. A., Bescos R. (2011). A comparison of fat mass and skeletal muscle mass estimation in male ultra-endurance athletes using bioelectrical impedance analysis and different anthropometric methods. *Nutricion hospitalaria*.

[15] Joyner M. J. (1993). Physiological limiting factors and distance Running. *Exercise and Sport Sciences Reviews*.

[16] Eston R., Reilly T. (2009). Kinanthropometry and Exercise Physiology Laboratory Manual. *Tests, Procedures and Data: Volume 1: Anthropometry*.

[17] Mitchell U. H., Bailey B., Owen P. J. (2020). Examining bone, muscle and fat in middle-aged long-term endurance runners: a cross-sectional study. *Journal of Clinical Medicine*.

[18] Szulc P., Duboeuf F., Chapurlat R. (2017). Age-Related Changes in Fat Mass and Distribution in Men--the Cross-Sectional STRAMBO Study. *Journal of Clinical Densitometry: The Official Journal of the International Society for Clinical Densitometry*.

[19] Jokl P., Sethi P. M., Cooper A. J. (2004). Master's performance in the New York City marathon 1983-1999. *British Journal of Sports Medicine*.

[20] Willy R. W., Paquette M. R. (2019). The physiology and biomechanics of the master runner. *Sports Medicine and Arthroscopy Review*.

[21] Brisswalter J., Nosaka K. (2013). Neuromuscular Factors Associated with Decline in Long-Distance Running Performance in Master Athletes. *Sports medicine*.

[22] Tanaka H., Seals D. R. (2003). Invited review: dynamic exercise performance in masters athletes: insight into the effects of primary human aging on physiological functional capacity. *Journal of applied physiology*.

[23] Vansant G., Van Gaal L., De Leeuw I. (1994). Assessment of Body Composition by Skinfold Anthropometry and Bioelectrical Impedance Technique: A Comparative Study. *Journal of Parenteral and Enteral Nutrition*.

[24] Lukaski H. C., Vega Diaz N., Talluri A., Nescolarde L. (2019). Classification of hydration in clinical conditions: indirect and direct approaches using bioimpedance. *Nutrients*.

[25] Nickerson B. S., Snarr R. L., Ryan G. A. (2020). Bias varies for bioimpedance analysis and skinfold technique when stratifying collegiate male athletes' fat-free mass hydration levels. *Applied Physiology, Nutrition, and Metabolism*.

[26] Sergi G., De Rui M., Stubbs B., Veronese N., Manzato E. (2017). Measurement of lean body mass using bioelectrical impedance analysis: a consideration of the pros and cons. *Aging Clinical and Experimental Research*.

[27] Silveira E. A., Barbosa L. S., Rodrigues A. P. S., Noll M., De Oliveira C. (2020). Body fat percentage assessment by skinfold equation, bioimpedance and densitometry in older adults. *Archives of Public Health*.

[28] Ripka W. L., Rotta C. V., Ulbricht L., Neves E. B. (2014). Body composition evaluated by skinfolds and bioimpedance in Brazilian men soldiers. *Revista Internacional de Medicina y Ciencias de la Actividad Fisica y del Deporte*.

[29] Sillanpää E., Häkkinen A., Nyman K. (2008). Body composition and fitness during strength and/or endurance training in older men. *Medicine and Science in Sports and Exercise*.

[30] Boughman J. K., Masters M. A., Morgan C. A., Ruden T. M., Rochelle S. G. (2019). Assessing the validity of bioelectrical impedance and skinfold calipers for measuring body composition in NOLS backcountry hikers. *Wilderness & Environmental Medicine*.

[31] Wells A. D., Bellovary B. N., Houck J. M. (2020). New multisite bioelectrical impedance device compared to hydrostatic weighing and skinfold body fat methods. *International Journal of Exercise Science*.

[32] de Abreu A. M., Wilvert L. C., Wazlawik E. (2020). Comparison of body mass index, skinfold thickness, and bioelectrical impedance analysis with dual-energy X-ray absorptiometry in hemodialysis patients. *Nutrition in Clinical Practice*.

[33] Leão C., Camões M., Clemente F. M. (2019). Anthropometric profile of soccer players as a determinant of position specificity and methodological issues of body composition estimation. *International Journal of Environmental Research and Public Health*.

[34] Vasold K. L., Parks A. C., Phelan D. M. L., Pontifex M. B., Pivarnik J. M. (2019). Reliability and validity of commercially available low-cost bioelectrical impedance analysis. *International Journal of Sport Nutrition and Exercise Metabolism*.

[35] Vitti A., Nikolaidis P. T., Villiger E., Onywera V., Knechtle B. (2020). The "New York City marathon": participation and performance trends of 1.2M runners during half-century. *Research in Sports Medicine*.

[36] Nikolaidis P. T., Cuk I., Clemente-Suárez V. J., Villiger E., Knechtle B. (2021). Number of finishers and performance of age group women and men in long-distance running: comparison among 10km, half-marathon and marathon races in Oslo. *Research in Sports Medicine*.

[37] https://www.athensauthenticmarathon.gr/site/index.php/en/results-en/491-results-2017-marathon.

[38] Knechtle B., Di Gangi S., Rust C. A., Nikolaidis P. T. (2020). Performance differences between the sexes in the Boston marathon from 1972 to 2017. *Journal of Strength and Conditioning Research*.

[39] Knechtle B., Barandun, Knechtle P. (2012). &nbsp;Running speed during training and percent body fat predict race time in recreational male marathoners. *Open access Journal of Sports Medicine*.

[40] Kasper A. M., Langan-Evans C., Hudson J. F. (2021). Come back skinfolds, all is forgiven: a narrative review of the efficacy of common body composition methods in applied sports practice. *Nutrients*.

[41] Lee S. Y., Gallagher D. (2008). Assessment methods in human body composition. *Current Opinion in Clinical Nutrition and Metabolic Care*.

